# Maintaining social capital in offenders with schizophrenia spectrum disorder—An explorative analysis of influential factors

**DOI:** 10.3389/fpsyt.2022.945732

**Published:** 2022-10-20

**Authors:** Lena A. Hofmann, Steffen Lau, Johannes Kirchebner

**Affiliations:** Department of Forensic Psychiatry, University Hospital of Psychiatry, University of Zurich, Zurich, Switzerland

**Keywords:** social capital, machine learning, schizophrenia, personal recovery, forensic psychiatry

## Abstract

The importance of “social capital” in offender rehabilitation has been well established: Stable family and community relationships offer practical assistance in the resettlement process after being released from custody and can serve as motivation for building a new sense of self off the criminal past, thus reducing the risk of re-offending. This also applies to offenders with severe mental disorders. The aim of this study was to identify factors that promote or hinder the establishment or maintenance of social relationships upon release from a court-ordered inpatient treatment using a modern statistical method—machine learning (ML)—on a dataset of 369 offenders with schizophrenia spectrum disorder (SSD). With an AUC of 0.73, support vector machines (SVM) outperformed all the other ML algorithms. The following factors were identified as most important for the outcome in respect of a successful re-integration into society: Social integration and living situation prior to the hospitalization, a low risk of re-offending at time of discharge from the institution, insight in the wrongfulness of the offense as well as into the underlying psychiatric illness and need for treatment, addressing future perspectives in psychotherapy, the improvement of antisocial behavior during treatment as well as a detention period of less than 1 year emerged as the most predictive out of over 500 variables in distinguishing patients who had a social network after discharge from those who did not. Surprisingly, neither severity and type of offense nor severity of the psychiatric illness proved to affect whether the patient had social contacts upon discharge or not. The fact that the majority of determinants which promote the maintenance of social contacts can be influenced by therapeutic interventions emphasizes the importance of the rehabilitative approach in forensic-psychiatric therapy.

## Introduction

Stable family relationships and community ties are known to have great potential in the prevention of re-offending among former inmates (1–3). In criminology, this potentially protective factor is also referred to as “social capital” (2–4). Broadly speaking, social capital can be defined as a person’s individual capacity to call upon personal ties and social networks in order to advance some personal interest and can provide former inmates with resources beneficial to their reintegration (e.g., employment, financial and emotional support, housing) (3, 5). Apart from the benefits of a social network regarding the individual quality of life, friends and family members may also serve as some sort of non-professional social control beyond the professional network, thus shaping behavior: A sufficient, high-quality social network improves treatment adherence and may reduce the risk of violating probation (6). In turn, involvement in a criminogenic peer group as well as social isolation elevate the risk for probation failures and violent behavior (7–9). Naturally, these considerations are not only important concerning the reintegration of offenders being discharged from the penal system but also for offender patients with psychiatric illnesses being discharged from institutional court mandated therapy. In fact, connectedness with others has been indicated as an important factor in personal recovery in various international forensic psychiatric populations as well as in a recent Swiss explorative study (10–12). With risk management being the core competence of the forensic psychiatrist, maintaining and promoting resources beneficial to the prevention of re-offending and to mental stabilization need to be targeted during the therapeutic process. However, offenders with mental illnesses often struggle to maintain social contacts through their inpatient treatment, or even to build one prior to the offense in the first place (10). The reasons for this are manifold: Relatives and other social contacts have to deal with both mental health issues and juridical problems and the consecutive stigma, they may be or have been the target of the patients’ aggressive behavior and may be subjected to ambiguous feelings regarding the patient (e.g., grief, disbelief, anger, guilt, shame) (13–15). Since their ability to establish and maintain social contacts is oftentimes critically impaired as a result of their illness, offender patients with mental disorders are all the more in need of therapeutic support to activate these resources during their treatment. Recently, several interventions to promote recovery in the sense of establishing a social network have been discussed and applied. An example for such a strength- and resource-based approach in forensic psychiatric therapy is the Good Lives Model, which focuses on building interpersonal skills and social networks in order to facilitate change in criminal behavior (16–18). However, due to the heterogeneity of forensic psychiatric patients, therapists need to be aware not only of the general factors that may impede social rehabilitation, but also of the individual parameters.

The present study aims to determine the most predictive factors of social capital upon discharge from court mandated inpatient treatment, based on a unique group of forensic offenders with schizophrenia spectrum disorder (SSD). To the authors’ knowledge, this is the first scientific evaluation of social capital in such a large homogenous population of offender patients with SSD.

## Materials and methods

The files of 370 delinquent patients diagnosed with SSD according to ICD-9 (295.x) (19) and ICD-10 (F20–29.x) (20), who were admitted to the Center for Inpatient Forensic Therapies of the University Hospital of Psychiatry Zurich for court mandated therapy, were assessed retrospectively. The case files consisted of professionally documented anamneses, psychiatric/psychologic inpatient and outpatient reports, police reports, testimonies, court proceedings, reports from social workers, and biannual extensive reports from clinicians as well as the nursing and care staff. Due to extensivity of the files and the high medical and legal importance assigned to cases of forensic patients in Switzerland, it can be assumed that the files contained all relevant information on the health and biography of a patient. A trained independent physician with + 5 years of forensic psychiatric experience systematically reviewed all case files and conducted a directed qualitative content analysis (21). A second trained independent rater encoded a random subsample of 10% of cases to assess inter-rater reliability. Cohen’s Kappa was 0.78, which can be regarded as substantial (22). The content analysis was performed according to a previously carefully designed questionnaire and rating protocol for coding based on a set of criteria originally proposed by Seifert (23–25). Before being put to use, questionnaire and extraction protocol design were repeatedly discussed in inter- and supervisions with senior researchers in forensic psychiatry from the researchers’ institution as well as other, international forensic psychiatric institutions.

The comprehensive dataset included items from the following domains: social-demographic data, childhood/youth experiences, psychiatric history, past criminal history, social/sexual functioning, details on the offense leading to forensic hospitalization, prison data, and particularities of the current hospitalization and psycho-pathological symptoms. The latter was defined by an adapted positive and negative syndrome scale (PANSS), whereby symptoms were divided into the usual 30 sub-categories and then rated on a three-tier scale instead of a seven-tier one (completely absent, discretely present, or substantially present). Social capital was defined as having at least one of the following social contacts: family ties, spousal relationships, a circle of friends of more than one person, and membership in a club. The dataset has already been evaluated in other studies as part of a larger, ongoing project with the goal of gaining further knowledge about the complex field of offender patients with SSD (7, 9, 26–35). Although the same database provides the basis for several analyses covering a wide range of objectives in this research area, and although there are a few overlapping parameters, it still contains a substantial number of unique variables, thus resulting in different theoretical and practical conclusions and implications. An overview of the basic characteristics of the population is provided in Table 1. Further details on data collection regarding our population can be found in Lau et al. (32).

**TABLE 1:** Sociodemographic characteristics.

Characteristics	Total n/N (%)	No social network n/N (%)	Social network n/N (%)
Male sex	339/369 (91.9)	125/140 (89.3)	214/229 (93.4)
Age at admission (mean, SD)	34.2 (10.4)	35.7 (10.9)	33.2 (9.7)
Native country Switzerland	140/369 (37.9)	57/140 (40.7)	110/229 (48)
Single (at offense)	244/363 (67.2)	93/140 (66.4)	151/223 (67.7)
Schizophrenia diagnosis	293/369 (79.4)	112/140 (80)	181/229 (79)
**Social network at discharge (multiple answers possible)**			
None	229/369 (62.1)		
Family	125/369 (33.9)		
Spouse	36/369 (9.8)		
More than one friend	24/369 (6.5)		
Member of a club	3/369 (0.8)		

SD, standard deviation; N, total study population; n, subgroup with characteristic.

**FIGURE 1:**
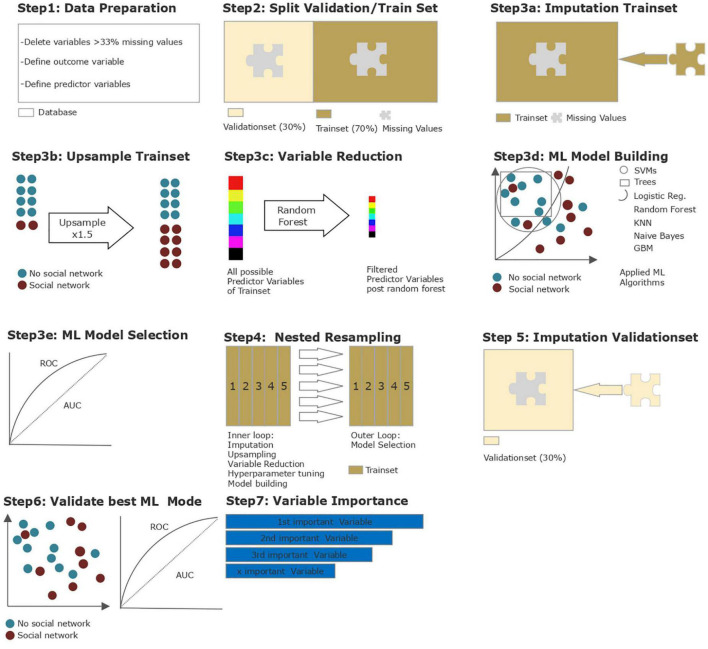
*Step 1—Data preparation*: Multiple categorical variables were converted to binary code. Continuous and ordinal variables were not manipulated. Outcome variable violent behavior/no violent behavior and 507 predictor variables were defined. *Step 2—Datasplitting*: Split into 70% training dataset and 30% validation dataset. *Step 3 a, b, c, d, e–Model building and testing on training data I*: Imputation by mean/mode; upsampling of outcome “social network” x1.5; variable reduction via random forest; model building via ML algorithms—logistic regression, trees, random forest, gradient boosting, KNN (k-nearest neighbor), support vector machines (SVM), and naive bayes; testing (selection) of best ML algorithm via ROC parameters. *Step 4—Model building and testing on training data II*: Nested resampling with imputation, upsampling, variable reduction and model building in inner loop and model testing on outer loop. *Step 5–Model building and testing on validation data I*: Imputation with stored weights from Step 3a and upsampling of outcome “social network” x2. *Step6—Model building and testing on validation data II:* Best model identified in Step 3e applied on imputed and balanced validation dataset and evaluated via ROC parameters. *Step 7—*Test for multicollinearity and ranking of variables by indicative power.

Parts of the following section were published beforehand in a study by Kirchebner et al. (30) and, as the same methodology was applied, are partly replicated here. For further information regarding data collection and processing, please refer to previous publications (30, 36). Due to the explorative nature of this study, supervised ML appeared to be the most suitable approach in identifying the most relevant predictive factors out of a large number of parameters and to determine the model providing the best predictive power. An overview of the statistical steps is shown in Figure 1 and is further described in detail below. All the steps were performed using R version 3.6.3. (R Project, Vienna, Austria) and the MLR package v2.171 (Bischl, Munich, Germany). CI calculations of the balanced accuracy were conducted using MATLAB R2019a (MATLAB and Statistics Toolbox Release 2012, The MathWorks, Inc., Natick, Massachusetts, United States) with the add-on “computing the posterior balanced accuracy” v1.0. All raw data were first processed for machine learning (see Figure 1, Step 1): Several categorical variables were converted to binary code, while continuous and ordinal variables were not adjusted. Due to the retrospective nature of the study and the large number of variables included, there were missing values among variables. This especially applied to information on the broader biographical history of patients, although forensic records were comprehensive. Variables with more than 33% missing values were eliminated, leaving a set of 508 variables. The outcome variable “social network upon discharge from the institution” was dichotomized into (a) “present” and (b) “not present.” Having social network referred to either having family or spouses, having more than one friend or being member of a social club. As there was missing data regarding their social network upon discharge in one case, leading to exclusion, a total of 369 patients remained. Out of all 369 patients, only 140 (37.9%) had some kind of social network upon their discharge, while 229 (62.1%) did not (see Table 1).

After data preparation, the database was divided into one training and one validation subset (see Figure 1, Step 2). The training subset, including 70% of all cases (*n* = 258), was used for variable reduction and model building/selection. To enable the flexible application of all ML algorithms, imputation of missing values was carried out and imputation weights saved for later were reused on the validation subset (see Figure 1, Step 3a). As the outcome variable was unevenly distributed, a random up-sampling at a rate of 1.5 was conducted, leading to a more balanced outcome (see Figure 1, Step 3b). A major objective of the present study was to identify the most important predictor variables from 508 possible variables. A decrease in variables can also counteract overfitting while maintaining computing times in initial model building at an acceptable level. Thus, we performed a variable reduction through random-ForestSRC down to the point where the AUC did improve by no more than 5% through adding another item (see Figure 1, Step 3c). This led to a variable reduction down to the 8 most important predictors. As the database was relatively small for ML purposes and our focus lay on variable extraction and prediction, we applied discriminative model building with logistic regression, trees, random forest, gradient boosting, KNN (k-nearest neighbor), support vector machines (SVM), and as an easily applicable generative model building, naive Bayes (see Figure 1, Step 3d). No hyperparameters were optimized. For each model, performance was calculated and assessed in terms of its balanced accuracy (the average of true positive and true negative rate, better suited for model evaluation and calculation of confidence intervals in imbalanced data) and goodness of fit (measured with the receiver operating characteristic, balanced curve area under the curve method, ROC balanced AUC). Specificity, sensitivity, positive predictive value (PPV), and negative predictive value (NPV) were also evaluated. As our training dataset was artificially balanced, the model with the highest AUC was chosen for final model validation with the validation subset (see Figure 1, Step 3e). To avoid dependencies between the variables, we tested the set of identified variables for multicollinearity. Finally, to prevent overfitting, a nested resampling approach was employed. For this purpose, we used a nested resampling model with the inner loop performing imputation, oversampling, variable filtration, and model building within 5-fold cross-validation, and the outer loop for performance evaluation also embedded in 5-fold cross-validation—a technique for artificially creating different subsamples of a dataset (see Figure 1, Step 4). To evaluate the model selected before, the validation subset with 30% of all cases (*n* = 111) was applied (see Figure 1, Steps 5–7). As briefly mentioned above, the previously stored imputation weights were reused on the validation subset (see Figure 1, Step 5). Then the selected model was applied for validation (see Figure 1, Step 6). The identified variables were finally tested for multicollinearity and ranked according to their indicative power (see Figure 1, Step 7).

## Results

### Model calculation

An overview of the performance parameters of the different calculated algorithms during the nested resampling procedure can be found in Table 2. With a balanced accuracy of 70% and an AUC of 0.77, the support vector machines algorithm (SVM) outperformed all the other ML algorithms. The absolute and relative distribution of the 8 most predictive variables identified during nested resampling and used for the model buildings are shown in Table 3. As described above, the model did not improve by adding another item. The quality of the final model in the validation step is provided in Table 4. As expected, the balanced accuracy of 64.7 and the AUC of 0.73 were lower than the results of the initial training model, but they were still meaningful. With a sensitivity of 50.9% and a specificity of 72.5%, patients holding some form of social capital upon discharge were identified correctly in half the cases, while three-fourths of cases were identified correctly as having no social capital (see Table 4).

**TABLE 2:** Machine learning models and performance in nested cross-validation.

Statistical procedure	Balanced accuracy (%)	AUC	Sensitivity (%)	Specificity (%)	PPV (%)	NPV (%)
Logistic regression	64.8	0.73	54.6	76.5	63.3	72.1
Tree	67.5	0.72	51.8	73.1	62.5	72.7
Random forest	68.2	0.74	59.9	76.5	64.3	72.8
Gradient boosting	66.1	0.75	55.9	76.3	64.3	70.8
KNN	62.4	0.72	66.4	58.3	52.3	72.2
**SVM**	**70**	**0.77**	**55.9**	**77.4**	**63.4**	**71.2**
Naive Bayes	70.9	0.75	65.7	76.3	67.1	76.3

AUC, area under the curve (level of discrimination); PPV, positive predictive value; NPV, negative predictive value; KNN, k-nearest neighbors; SVM, support vector machines. machines. The bold values represent the performance values of the SVM algorithm as most suitable algorithm.

**TABLE 3:** Absolut and relative distribution of relevant predictor variables.

Variable code	Variable description	Social network	No social network
S1	Social isolation at time of offense	90/138 (65.2)	**120/144 (83**.3)
S8e	Living situation at time of offense: at parents/mother/father	**51/148 (34.5)**	27/183 (14.8)
R15g	Main content of psychotherapy: future perspectives	**72/162 (44.4)**	38/191 (19.9)
R19	Insight into wrongfulness of offense during current hospitalization	**103/153 (67.3)**	64/185 (34.6)
R26	Insight into illness and its treatment	**88/144 (61.1)**	44/146 (30.1)
R27b	Improvement antisocial behavior during treatment	**140/136 (85.9)**	137/192 (71.3)
J1	Time spent in prison > 1 year	45/155 (29)	**64/178** (36)
R28	Legal prognosis at discharge		
	Favorable	**52/150 (34.7)**	20/161 (12.4)
	Sufficient	**42/150** (28)	36/161 (22.4)
	Doubtful	23/150 (15.3)	**39/161 (24.2)**
	Unfavorable	33/150 (22)	**66/161** (41)

SD, Standard deviation; PANSS, positive and negative syndrome scale. The bold values represent the two subgroups expressed each predictor variable (e. g., patients with no social network upon discharge were socially isolated at the time of the offence, while patients with a social network upon discharge had been previously living with their parents at the time of the offence).

**TABLE 4:** Final SVM model performance measures.

Performance measures	% (95% CI)
Balanced Accuracy	64.7 (56.3–72.8)
AUC	0.73 (0.64-0.83)
Sensitivity	50.9 (50.5-51.3)
Specificity	79.6 (79.3-80.0)
PPV	72.5 (72.1-72.9)
NPV	60.6 (60.2-60.9)

### Predictors of a social network upon discharge

**FIGURE 2:**
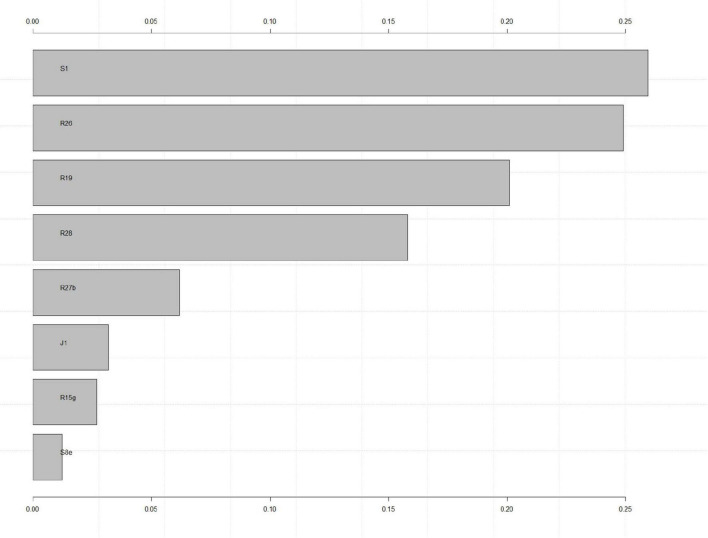
S1, social isolation at time of offense; R26, insight into illness and its treatment; R19, insight in wrongfulness of offense during current hospitalization; R28, assigned legal prognosis at discharge; R27b, improvement of antisocial behavior during treatment; R15g, main content of psychotherapy: future perspectives; J1, time spent in prison >1 year; S8e, Living situation at time of offense: at parents/mother/father.

The distribution of the importance of variables of the final validation model is presented in Figure 2 as a one-sided tornado graph. *Social isolation at time of the offense committed*, a *time spent in prison for more than a year* and a *doubtful or unfavorable legal prognosis* emerged as most indicative factors for having no social network upon discharge, while *insight into the illness and treatment*, developing *insight into the wrongfulness of the committed offense* during treatment, a *sufficient or favorable legal prognosis*, an *improvement of antisocial behavior*, and *living together with parents/mother/father at the time of the offense* were most indicative for having a social network upon discharge. Another predictive factor that proved beneficial in the presence of a social network upon discharge was a *psychotherapeutic focus on future perspectives*.

## Discussion

The aim of this study was to determine the factors that distinguish between offender patients with SSD who had some form of social capital upon discharge from court mandated inpatient treatment from those who did not. The goal was to use an explorative approach to identify the most predictive factors in social contacts and community ties upon discharge. In order to do so, we applied ML algorithms to a large database consisting of 369 patients and were thus able to create an appropriate model. With a balanced accuracy of 65% and an AUC of 0.73, the SVM model could correctly identify patients without social capital upon their discharge in three-quarters the cases. Variables related mostly to life conditions before the patients’ detention, the decline in socially intolerant behavior due to either illness or antisocial behavior as well as to the estimated risk of re-offending after discharge. A longer *duration of imprisonment (*>*1 year)* was also identified as negative predictor of social ties upon discharge. This seems obvious: Establishing a social network is already difficult for patients with SSD due to their symptomatology, and is further exacerbated by institutionalization, which is usually accompanied, at least initially, by strict regulation of access to the outside world (including digital communication such as via social media). It seems therefore understandable, that the formation of community ties is severely impaired due to their often year-long hospitalization. This is in line with findings regarding offenders without mental disorders who have been subjected to long imprisonment (37–39). It appears that despite forensic-psychiatric treatment focusing on rehabilitation, it failed to establish a social network in those cases. This fact raises even more concern as it has been suggested that social contacts and support may in turn contribute to a shortened duration of the institutionalization in forensic psychiatric populations (40). However, the findings on that matter are yet contradictory and, in a recent Swiss study, lack of social contacts has not been confirmed as predictor for a longer duration of stay in the specific group of offender patients with SSD (29, 41). Two overlapping static predictors of social capital were general *social isolation at the time of the offense*, which was associated with a lack of social contacts upon discharge in three-fourths of cases, and *living situation at the time of the offense*: Patients who had been living together with both or one parent prior to their institutionalization could rely on some form of social network upon discharge at least in around one-third of cases. As the social network upon discharge comprised mostly of family members rather than friends, spouses or communal ties (see Table 1), it seems understandable that a previously existing family relationship made it more likely for patients to reintegrate into after their discharge from the institution. This emphasizes the importance of integrating and involving relatives in the therapeutic process: In doing so, clinicians and therapists can help reduce the double stigma of patients and their families (mental illness and criminal offense/internment measure), empower family systems through active participation and improved psychoeducational knowledge transfer, and create transparency in the therapeutic process. Research on the need and burdens of relatives of mentally ill offenders is still limited, but the few existing findings clearly indicate family members’ need for participation (13, 42). In line with the scarcity of research in this area, there are hardly any established, operationalized interventions for the involvement of relatives in forensic psychiatric therapy. Nevertheless, a turnaround has become apparent in the last few years, which is mainly driven by family members’ initiatives and projects (43, 44).

Further predictors related primarily to the patients’ development during the therapeutic process: *Insight into the illness and its treatment* and the *improvement of antisocial behavior during treatment* emerged as highly relevant predictors for having social capital upon discharge. This could be well expected, as both symptomatology of SSD as well as antisocial behavior and attitudes impair social compatibility. If it is assumed that a certain degree of antisociality is also an expression of SSD in offender patients in the sense of a loss of the set of values and norms, there is also a high degree of interdependence between the two items and by targeting the former through therapeutic interventions, the latter is positively influenced as well. On the other hand, the possibility of comorbid antisocial behavior patterns in patients with SSD, especially those with involvement in the judicial system, has been discussed as well (45). Either way, these findings highlight the importance of targeting both the underlying SSD through psychoeducational measures as well as antisocial attitudes and behavior, e.g., through group therapy programs on social skills as well as standardized treatment programs such as the cognitive-behavioral Reasoning and Rehabilitation Programme, which was originally developed for use in the correctional system, but has been further developed for the rehabilitation of offenders with mental illness (46). Furthermore, insight into the illness and its treatment forms the basis for many other factors that positively influence the further course: insight into the need for treatment correlates with treatment adherence, which in turn positively influences clinical outcome regarding current symptomatology as well as psychotic relapses and chronification, hospitalization rates and re-offending rates (47–51).

Another dynamic factor predicting the presence of social capital upon discharge was the patients’ *insight into wrongfulness of the offense*. This result could be interpreted in two ways: It may be possible that patients who are socially connected are more often confronted by their relatives and network regarding the offense leading to the institutionalization. This may be especially the case for patients whose victims belong to their social network, e.g., family members. Another plausible explanation for this finding is that patients who have the capacity to develop and maintain social capital are in general healthier and less impaired by their underlying mental illness than their socially isolated counterparts, and that those patients are therefore also more capable of actually reflecting on their behavior and past actions. It is also possible that, while having actually little meaningfulness when it comes to re-delinquency, a previously existing social network may be more willing to keep in contact with offender patients if they critically examine and reflect upon their actions, as it is often demanded by the public, the media and society in general (52).

The *legal prognosis assigned at release*, i.e., the risk of re-offending, also played a role in the development and/or maintenance of social contacts: Patients with a rather unfavorable or doubtful legal prognosis could rely on a social network upon discharge less often than patients with a lower risk of re-offending. It could be hypothesized that the risk of re-offending is not so much causal for the existence of a social network at the time of discharge, but rather that it is the other way around: Clinicians and forensic psychiatric experts evaluate the risk of re-offending constantly during the course of the institutionalization as well as prior to the patients’ release, based on various established risk assessment tools, which often include the presence of social contacts as a protective factor (e.g., SAPROF) (53, 54). In fact, successful cooperation with relatives or other social contacts during the therapeutic process is associated with lower rates of criminal recidivism (55). Just as with insight into wrongfulness of the offense, another explanation for this finding may very well be that healthier patients are labeled as less likely to re-offend and are also more capable of maintaining social contacts.

Finally, patients were more likely to have some sort of social capital if the *main psychotherapeutic content was future perspectives*. Yet, this item did not exclusively refer to the current or future social network, but could include ideas on housing, occupational and financial perspectives as well. This fits well with findings from non-offending patients with SSD highlighting the importance of an encouraging psychotherapy as part of the recovery process (56). However, to the knowledge of the authors, the content of psychotherapeutic sessions regarding future perspectives has not specifically been explored in research yet.

Looking at the predictors identified above, a considerable overlap between many factors is striking: Naturally, forensic psychiatric experts assign a more favorable legal prognosis to patients with more insight into their illness and the wrongfulness of their index offense, and with a more stable treatment adherence, which often stems from insight into the necessity of treatment. Patients with such insight are also more likely to actively engage in constructive psychotherapy and may be more open toward the development of realistic future perspectives. Accordingly, the predictors of the presence of a social network at discharge cannot be viewed as singular factors acting independently of one another. Rather, they must be regarded as a mutually dependent structure. Interestingly, these predictors even dominated factors related to the severity of the disorder upon discharge (e.g., positive and negative symptoms according to the PANSS) or the severity of the offense previously committed. Insight into the illness and the consecutive need for treatment, reflected in treatment adherence, has also been associated with recovery in non-offender patients with SSD (56, 57). However, it has to be noted that insight into one’s own illness and need for treatment can be a double-edged sword: Studies in non-forensic populations of patients with SSD have shown that those who accept they are suffering from a mental illness often feel disempowered and stigmatized, are more likely to adopt a “disabled role” and to socially withdraw (58–60). This can of course hinder the recovery process. Similar observations have not yet been made in forensic psychiatry, and should be explored further in the future, especially as offenders with SSD may be subjected to the double stigmatization of being mentally ill and having a history of delinquency.

Considering limitations of the present study, the retrospective approach is of course notable. Accordingly, the parameters studied were not collected continuously in a standardized manner comparable to a prospective study design. This is not so much a problem for clearly defined variables, such as the length of the prison sentence, but poses difficulty for variables with a certain degree of latitude in their definition (e.g., antisocial behavior or insight in illness), as they may be subjected to a higher interrater bias. Even though interrater reliability was sustainable, as described in the methodology section, one has to be careful as to draw conclusions from especially the dynamic predictors and their clinical implications. While for instance the focus on future perspectives in psychotherapy was found to be of influence regarding social contacts, it was not clearly defined as to how exactly future perspectives were targeted (e.g., whether they related to occupational, financial or social aspects). The authors therefore recommend a replication of the current findings in a prospective setting to evaluate the robustness of causal inferences drawn from them. Furthermore, while populations in forensic psychiatric research are often relatively small compared to general psychiatry and other medical specialties, it has to be acknowledged that our sample of 369 patients, all collected from one single institution, can merely serve an exploratory purpose. Further application of the model to larger populations and other institutions is therefore recommended. As our population consisted only of offender patients with SSD, it has to be noted that the results presented may not be applied to other offender populations (e.g., with personality disorders, offenders with no psychiatric disorder). Also, as it would be expected in an offender population, there were only few women in our sample. This may limit the generalizability to female offender populations. Another notable aspect is that the focus in this study is exclusively on the presence or absence of a social network after discharge. Of course, social contacts are merely one facet of the complex concept of personal recovery. While it is well established how social capital benefits reintegration in society of mentally ill offenders, it is not possible to infer the actual importance of the social network at discharge in the overall construct of personal recovery from the present results. Future research on that matter is therefore needed. Finally, the study design allowed only an explorative variable reduction—although it was possible to break down over 500 variables to the 8 most influential, the findings do not allow a prospective model building regarding social reintegration. Lastly, when critically evaluating the process of model building, it has to be noted that the uneven distribution of the outcome variable would have had negative impact on the model building process and had to be counteracted through up-sampling. This however was performed merely on the training set during the model building process with the goal of creating optimal “lab conditions.” As the validation set did not undergo up-sampling, this procedure does not interfere with the validity of the results. The lack of up-sampling and the resulting uneven distribution of the outcome variable in the validation set also explains why the SVM performed with better performance parameters in the training set under artificially created ideal conditions.

In summation, the present findings contribute to a better understanding of social rehabilitation of offender patients with SSD after court mandated inpatient therapy. Through the use of ML as modern statistic instrument, we were able to identify the 8 factors most related with social capital upon discharge as well as their complex interplay out of a large dataset with over 500 different parameters. Clinical implications here are most likely to arise from the dynamic parameters that can be influenced by therapeutic interventions during hospitalization, whereas the static parameters that cannot be influenced by the course of treatment (e.g., social isolation at the time of the offense) might highlight special needs of affected patients. Of course, these findings do not allow other factors known to influence social reintegration to be dismissed as unimportant. As stated in the discussion of the limitations, social reintegration and recovery are complex processes which cannot be comprehensively evaluated with one single exploratory analysis. However, the findings highlight the value and importance of focusing on social resources and factors promoting them (e.g., decrease of antisocial behavior). Further research on the subject is needed, ideally in prospective trials considering various aspects of social re-integration and the maintenance of the social capital gained.

## Data availability statement

The raw data supporting the conclusions of this article will be made available by the authors, without undue reservation.

## Ethics statement

The studies involving human participants were reviewed and approved by Ethics Committee of the Canton of Zurich. Written informed consent for participation was not required for this study in accordance with the national legislation and the institutional requirements.

## Author contributions

JK: conceptualization, methodology, software, and data curation. JK and LH: validation, formal analysis, and investigation. LH, JK, and SL: resources and writing—review and editing. LH: writing—original draft preparation and visualization. SL and JK: supervision. SL: project administration. All authors have read and agreed to the published version of the manuscript.
